# Existence of a novel *qepA* variant in quinolone resistant *Escherichia coli* from aquatic habitats of Bangladesh

**DOI:** 10.1186/s13099-017-0207-8

**Published:** 2017-10-23

**Authors:** Zillur Rahman, Aminul Islam, Mahamud-ur Rashid, Fatema-Tuz Johura, Shirajum Monira, Haruo Watanabe, Niyaz Ahmed, Andrew Camilli, Munirul Alam

**Affiliations:** 1 International Center for Diarrhoeal Disease Research, Bangladesh (icddr,b), Mohakhali, Dhaka, 1212 Bangladesh; 2 Department of Bacteriology, National Institute of Infectious Diseases, Tokyo, Japan; 3 Department of Molecular Biology and Microbiology, Howard Hughes Medical Institute, Tufts University School of Medicine, Boston, USA

**Keywords:** Novel *qepA* variant, *qepA4*, Quinolone resistance, *Escherichia coli*, Bangladesh

## Abstract

Of 19 environmental *Escherichia coli* (n = 12) and *Klebsiella pneumoniae* (n = 7) tested for quinolone resistance-related genes *qnrA*, *qnrB*, *qnrC*, *qnrS* and *qepA*, four each of *E. coli* and *K. pneumoniae* possessed *qnrS*, and another *E. coli* isolate possessed a new variant of *qepA*. This is the first detection of *qepA* in environmentally dwelling bacteria in Bangladesh.

## Background

Acquisition of resistance towards several classes of antibiotics and the spread of multi-drug resistant bacteria is of immense public health concern globally. Increased use of quinolones, a family of synthetic chemical agents and one of the most commonly prescribed broad-spectrum antimicrobials, in recent years have coincided with the increasing incidence of resistance to this drug among *Enterobacteriaceae*. For years, quinolone resistance was believed to be solely chromosomally mediated through nucleotide substitutions in the quinolone resistance-determining region of DNA gyrase (*gyrA* and *gyrB*) and topoisomerase IV (*parC* and *parE*), which are the main target molecules of the quinolones [1]. However, plasmid-mediated quinolone resistance (PMQR) was subsequently discovered. The PMQR gene, *qnrA* (currently *qnrA1*), encoding a protein that protects type II topoisomerases from quinolones, was first reported in 1998 [2]. Thereafter, as many as six Qnr families, named Qnr A, B, C, D, S and VC, were identified [3]. Another reported mechanism of PMQR is the covalent modification of certain quinolones including ciprofloxacin by a plasmid-encoded variant of aminoglycoside acetyltransferase AAC (6′)-Ib-cr [4]. A third mechanism of PMQR known since 2007 is efflux of quinolones by the efflux pump-encoding genes, *qepA* and *oqxAB* [5, 6].

QepA was found to be associated with decreasing susceptibility, and 8 to 32-fold increase of minimal inhibitory concentration values of the hydrophilic quinolones such as norfloxacin, ciprofloxacin and enrofloxacin [5]. So far, three variants of *qepA* named *qepA1*, *qepA2* and *qepA3* have been reported [5, 7, 8]. Although PMQR determinants confer a low-level of resistance to quinolones, they provide a favorable background for the selection of additional chromosome-encoded quinolone resistance mechanisms, which make clinical therapy more difficult [9]. All of these PMQR determinants are increasingly being identified worldwide in members of the *Enterobacteriaceae.* Since aquatic environment serves as a reservoir for diverse microbial populations including bacteria carrying PMQR determinants [13], selection pressure from residual antibiotics might facilitate horizontal transfer of these quinolone resistance genes to pathogenic bacteria. The present study was designed to investigate the presence of quinolone resistance related genes *qnrA*, *qnrB*, *qnrC*, *qnrS* and *qepA* in members of the family *Enterobacteriaceae*, namely *E. coli* and *K. pneumoniae*, isolated from a lake and a river of Dhaka city, Bangladesh. Both bacteria demonstrated frequent presence of *qnrS*, and one of the *E. coli* isolates possessed a new sequence variant of *qepA* designated as *qepA4.*

## Methods

Twelve *E. coli* and seven *K. pneumoniae* were isolated from water samples collected from a lake and a river of Dhaka city. After preliminary identification by standard culturing methods, all isolates were confirmed using API 20 E (bioMérieux, France) strips. Antimicrobial susceptibility was determined by the Kirby-Bauer disk diffusion method according to the Clinical Laboratory Standards Institute recommendations. Commercially available antibiotic discs (Oxoid) of nalidixic acid (30 µg), ciprofloxacin (5 µg) and levofloxacin (5 µg) were used in the antibiotic susceptibility assay. Bacterial DNA was obtained from all isolates by the standard boiling method. Detection of PMQR genes (*qnrC*, *qepA*, *qnrA*, *qnrB*, *qnrS* and *rmtB*) was performed by polymerase chain reaction (PCR) using the primers as described elsewhere [10, 11]. In addition we designed three pair of primers.

(F1-CCTGAATGCGGATGGGTGT, R1-TGTCCAGGATCCAGAGAAGC;

F2-GCGTGTTGCTGGAGTTCTTC, R2-GCTGAATTCGGACACCGTCT;

F3-TGTTCACCATCGGCAACGA, R3-CACCTTCACCAAGACCACG; all listed 5′–3′) targeting different regions of *qepA* to get the full sequence (1536 bp) using overlapping PCR amplicons. The amplified fragments of *qepA* were then sequenced using an ABI PRISM BigDye Terminator Cycle Sequencing Reaction kit (Applied Biosystems) on an ABI PRISM 310 automated sequencer (Applied Biosystems). The deduced full length sequence of the gene was submitted to GenBank (Accession Number KX580704).

## Results and discussion

Nearly half of the isolates in the present study were found to be resistant to quinolone antibiotics such as nalidixic acid (52.6%), ciprofloxacin (47.4%), and levofloxacin (47.4%). Among the 19 isolates, ten were resistant to at least one of the tested quinolones. Of the remaining nine isolates, four showed intermediate resistance to nalidixic acid, while one of the ten quinolone-resistant isolates exhibited intermediate resistance to levofloxacin. As quinolones are broad-spectrum antibiotics, people use them for wide ranging infections in Bangladesh. Morgan et al. [12] reported a frequency of 86% non-prescriptional antibiotic use in Bangladesh. Our data are suggestive of evolutionary response to this indiscriminate use of quinolones in the form of an alarmingly high incidence of quinolone resistance among the members of the family *Enterobacteriaceae*.

All the isolates (n = 19) were screened for PMQR genes by PCR. Of these, four each of *E. coli* and *K. pneumoniae* possessed *qnrS*, while another *E. coli* isolate possessed *qepA*. Unlike our study, most of the previous studies were based on clinical isolates and the most frequent PMQR gene hitherto identified has been *qnrB* [9]. However, this gene was not detected in any of the bacteria tested in our study. Also, none of the other two PMQR genes, *qnrA* and *qnrC*, were found in any of the isolates tested here. Five isolates carrying *qnrS* were found sensitive to all three quinolone antibiotics tested. Although strains carrying this gene did not exhibit resistance to the tested quinolone drugs, it is possible that *qnrS* might play a role in the resistance to other quinolones. Nonetheless, an environmental reservoir for the quinolone resistance related genes was demonstrated in the present study, supporting the results of an earlier study [13], which would allow horizontal transfer of gene(s) responsible for resistance to quinolone antibiotics.

QepA, a recently identified PMQR gene encoding an efflux pump [5], was detected in one of the *E. coli* isolates resistant to all three quinolone antibiotics tested in the present study. To our knowledge, this is the first time in Bangladesh that a *qepA* gene was found from environmentally occurring bacteria belonging to the family *Enterobacteriaceae*. The association of *qepA* with the *rmtB* gene contributing to high level resistance to aminoglycosides was reported from Belgium, China and Japan [5, 14, 15]. Previous studies on *E. coli* showed an association of *qepA* and *rmtB* with a composite transposable element flanked by two copies of IS*26* [5]. In our study, the *E. coli* isolate possessing *qepA* did not have *rmtB*, as confirmed by PCR. This result suggests the possibility of independent emergence of *E. coli* harboring *qepA* locally in the aquatic environment, and possibly of different ancestry from the ones carrying *qepA* and *rmtB*, as reported from other countries [5, 14].

**Fig. 1:**
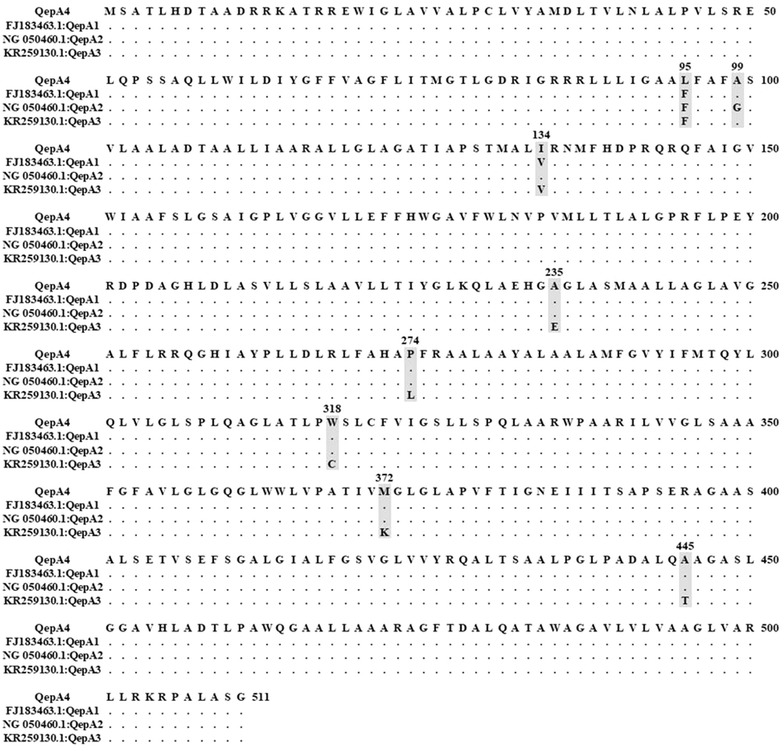
CLUSTAL W alignment of QepA1, QepA2, QepA3 and QepA4 from isolate E1F. Dot (.) indicates identical sequences

**Fig. 2:**
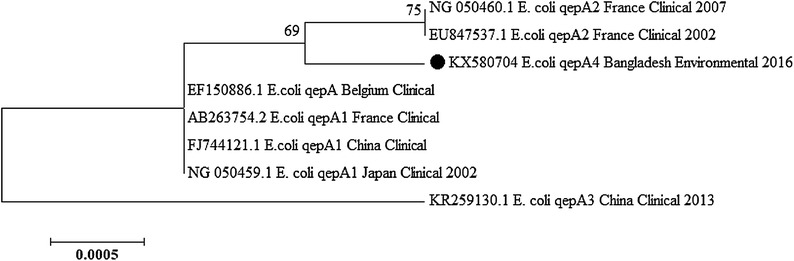
Phylogenetic tree constructed by the neighbour-joining method by MEGA 6.0 based on the *qepA* gene isolated from different locations worldwide. Numbers at the tip of branches denote the bootstrap percentages for 1000 replicates. Evolutionary distance of the nucleotide substitutions per site is indicated by the scale

In the present study, DNA sequencing of *qepA* revealed the gene to be different from the three alleles of *qepA* (*qepA1*, *qepA2* and *qepA3*) that have been reported so far [5, 7, 8]. Comparison of the 1536 bp DNA sequence of *qepA* (Accession Number KX580704) revealed eight nucleotide differences that result in eight amino acid substitutions in the deduced 511 bp amino acid sequence (Fig. 1). Thus, this *qepA* gene from environmental *E. coli* isolate E1F represents a new variant of the gene, and was designated *qepA4*. A phylogenetic tree constructed by the neighbor joining method (MEGA 6.0) revealed separation of *qepA4* from all *qepA* alleles reported so far, although it is most closely related to the *qepA2* branch (Fig. 2). This result, combined with the absence of a linked *rmtB* gene noted above, suggests a different lineage for *E. coli* carrying *qepA* found in Bangladesh. This novel variant of a quinolone resistance determinant and the presence of other PMQR genes in naturally disseminating *Enterobacteriaceae* call for urgent surveillance to monitor and prevent the spread of these resistant bacteria in vulnerable populations of Bangladesh.
